# Hyaluronic acid compared with corticosteroid injections for the treatment of osteoarthritis of the knee: a randomized control trail

**DOI:** 10.1186/s40064-016-2020-0

**Published:** 2016-04-12

**Authors:** Alireza Askari, Tahereh Gholami, Mohammad Mehdi NaghiZadeh, Mojtaba Farjam, Seyed Amin Kouhpayeh, Zahra Shahabfard

**Affiliations:** Department of Orthopedics, School of Medicine, Shiraz University of Medical Sciences, Shiraz, Iran; School of Public Health, Fasa University of Medical Sciences, Fasa, Iran; Department of Community Medicine and Statistics, Fasa University of Medical Sciences, Fasa, Iran; Non-communicable Diseases Research Center, Fasa University of Medical Sciences, Fasa, Iran; Department of Pharmacology, Fasa University of Medical Science, Fasa, Iran; School of Nursing, Shiraz University of Medical Sciences, Shiraz, Iran

**Keywords:** Osteoarthritis, Intra-articular injection, Hyaluronic acid, Corticosteroids, Pain, Stiffness, Iran

## Abstract

**Background:**

Osteoarthritis (OA) is the most common chronic condition of the joints that takes place when the cartilage or a low friction surface between joints breaks down which leads to pain, stiffness and swelling. The purpose of the present study was to evaluate the therapeutic effect of intra-articular hyaluronic acid (HA) in comparison to corticosteroids (CS) for knee osteoarthritis.

**Methods:**

140 patients with knee osteoarthritis, who were followed for 3 months, were randomized to receive intra-articular injection of either hyaluronic acid or corticosteroid. By receiving one injection of drug during the enrollment in the study, the patients were treated. With the Western Ontario and McMaster University Osteoarthritis Index (WOMAC), Knee injury and Osteoarthritis Outcome Score (KOOS), and the visual analog pain scale, an independent, blinded evaluator assessed the patients three times.

**Results:**

The mean age of the patients in the corticosteroid group were 57 ± 1.9 years and in Hyaluronic acid group were 58.5 ± 8.3 years. WOMAC score represented that pain and stiffness did not improve in neither groups at any time points after intervention (P > 0.05). KOOS score suggested that symptoms improved after 3 months in both CS and HA groups. Besides, daily activity improved in both groups (P < 0.05).

**Conclusions:**

As a conclusion, it is argued that the most important difference between the two intervention groups is the duration of effectiveness. HA is suggested to be superior in the duration of pain relief when compared to CS. We can propose that HA can be administered every 3 months intra-articular for knee joint OA. Therefore, when CS has to be injected every 2 months, it will be more convenient to use HA.

## Background

Osteoarthritis is a chronic progressive joint disease which often affects middle-age to the elderly (Lawrence et al. 2008). A recent finding showed that symptomatic knee OA occurs in 10 % of men and 13 % of women aged 60 years or older (Zhang and Jordan 2010). The research demonstrated that multifactorial etiology such as a variety of risk factors including aging, genetics, trauma, mal alignment, and obesity interact with one another to cause the mentioned disorder (Loeser 2001). Pain reduction, joint mobility improvement, and functional impairment limitation are the major objectives in OA treatment. Moreover, regarding the maintenance of patients’ independence and quality of life, secondary goals have been taken into account for the reduction of disease progression and improvement of muscular strength (Snibbe and Gambardella 2005). There are various conservative treatments for knee OA which provide short-term effectiveness with their own advantages and disadvantages (McArthur et al. 2012). By using a variety of non-surgical treatments, such as oral analgesics, non-steroidal anti-inflammatory drugs, or any of several types of intra-articular injections, for most patients it might prolong time to undergo surgery. This would be true if there could be any disease- modifying medication. This is why hyaluronic acid (HA) and corticosteroids (CS) are of particular interest.

Not merely intra-articular HA injections have a beneficial effect in the treatment of OA but also has some integral roles in improving joint lubrication, synovial fluid viscosity, normalizing hyaluronan synthesis, inhibiting proteoglycan degradation, exhibiting analgesic and anti-inflammatory effects (Day et al. 2004). Additionally, it has a long duration of action (Dixon et al. 1988; Dougados et al. 1993).

In recent decades, intra-articular injections of CS have been used in the treatment of OA but clinical evidences suggest short-term effectiveness, usually one to 4 weeks. Long-term treatment could promote joint destruction and tissue atrophy (Raynauld et al. 2003). Studies of cartilage damage, however, tend to recommend that changes are more likely due to the underlying disease than the steroid injection (Ayral 2001). Several clinical studies have been compared face to face HA and CS in knee OA (Leighton et al. 2014; Shimizu et al. 2010; Skwara et al. 2009). Colen et al. (2010), in meta-analysis study compared HA and CS and provided an efficacy pattern which had been changed by the passage of time and came into this conclusion that 8 weeks after injection, HA had greater efficacy. Numerous systematic reviews have investigated HA effects and other placebos (Colen et al. 2012; Divine et al. 2007), or CS and placebos (Arroll and Goodyear-Smith 2004; Godwin and Dawes 2004), however, there are a number of studies about HA and CS (Bannuru et al. 2009). Thus, the aim of the present study was to determine which treatment method was more effective to compare intra-articular HA injection with CS.

## Methods

### Design

This was a randomized, double blind study with parallel groups. After approval of institutional research deputy and ethics committee, the patient suffering from knee OA were randomized to receive IA injections of 2 cc of high molecular weight (500,000–730,000) HA (Fidia Farmaceutici S.p.A, Italy) or IA injections of 40 mg CS. The treatment consisted of one IA injections of HA or one injection of CS. And the follow-up visits were scheduled at 1, 2, and 3 months. Before inclusion, for fulfillment of the entry criteria, patients were evaluated. Moreover, qualified patients were informed about the aim and design of the study.

Adequate patients were randomized 1:1. A computer-generated list of random numbers was used. The random sequence was created through the freely accessible tools available at http://www.randomization.com, which uses the pseudo-random number generator of Wichmann and Hill (1982) modified by (McLeod 1985). This method helps to introduce several aim of interventions which includes a basis for the generator of random number which allows reproduction of the randomization model of one study when details and labels are assigned similarly.

At the initial visit the allocation sequence was hidden from the people in order to determine the patient’s eligibility. When the patients’ eligibility was confirmed by a physician, a number was presented. According to the randomization list, a physician was responsible for the patients’ assignment. Both the researcher in charge of evaluations at follow-up and patients’ group assignment and the patients were blind. By inserting the needle into the suprapatellar pouch, under aseptic conditions, administration of the IA treatments occurred using a single injection, planned to be repeated at 3 months. To avoid any effusions, before each injection, Arthrocentesis was performed.

### Patients selection criteria

Qualified patients included men and women from 45 to 80 years who were suffering from knee OA for at least 3 months, along with radiographic OA grade II–III (According to Kellgren and Lawrence (KL) grading scale (Kellegren and Lawrence 1957), who signed the informed agreement form for participation). Main excluded participants for this study included a history or presence of trauma or surgery or cancer or malignant tumors, infections and sores on the target knee, history of vasovagal shock, use of NSAIDs in 2 days prior to injection, any receiving corticosteroids injection in the knee in the last 6 months, pregnancy and lactation.

### Instruments

#### Western Ontario McMaster University Osteoarthritis (WOMAC)

In this study, Farsi version of the WOMAC index was used (Nadrian et al. 2012). The index is a 24-item questionnaire divided into three subscales which measure pain (5 items, score range 0–20), stiffness (2 items, score range 0–8), and physical function (17 items, score range 0–68). The three normalized subscale values were summed to provide the normalized WOMAC-total score. In the last section of follow-up, the researcher asked the WOMAC questions again.

#### The Knee Injury and Osteoarthritis Outcome Score (KOOS)

Having designed to evaluate the patients’ attitude on the knees and related problems, the KOOS is a tool specifically used for knee. The KOOS measures not only short-term but long-term sequellae of knee injury. It contains 42 items in 5 scored subscales including Pain, Other Symptoms, Function in daily living (ADL), Function in Sport and Recreation (Sport/Rec), and Knee-related Quality of Life (QOL) (Roos and Lohmander 2003).

#### Visual analog scale

The visual analog scale (VAS) is an instrument regularly used to measure pain intensity based on a 0–10 cm (Flandry et al. 1991). In the present study, the researcher asked the patients: “Based on VAS, how much pain are you in/experiencing?” In the follow-up sections, based on VAS, the researcher asks about their pain again. The measurement was recorded by the orthopedic surgeon.

### Statistical analysis

Data was presented as mean ± standard deviation. Comparison of demographic variables were done with Chi square test. VAS pain, WOMAC and KOOS sub scores was evaluated during time and simultaneously compared between study groups with repeated measurement of ANOVA. All statistical analysis were done in IBM SPSS 19 (SPSS Inc, Chicago, IL). P value < 0.05 considered as significant level.

## Results

In this study 69 patients at corticosteroid group with age of 57.0 ± 9.1 years and 71 in Hylan group with 58.5 ± 8.3 years were participated (P = 0.322). In corticosteroid group 17.4 % and in Hylan group 12.7 % were male (P = 0.435). Table 1 presented other characteristics of the groups. Two groups were at same marital (P = 0.984), education (P = 0.984) and occupation status (P = 708), and cigarette smoking (P = 0.984). All 140 recruited patients complete the 3 months follow-up.

**Table 1:** Baseline demographic information

	Corticosteroid		Hylan		P value Chi square
	n	%	n	%	
Sex					
Male	12	17.4	9	12.7	0.435
Female	57	82.6	62	87.3	
Occupation					
Housekeeper	55	79.7	56	78.9	
Retired	2	2.9	4	5.6	0.708
Occupied	12	17.4	11	15.5	
Education					
Less than High School Diploma	54	78.3	53	74.6	0.615
High School Diploma or More	15	21.7	18	25.4	
Marital					
Live together	68	98.6	70	98.6	0.984
Live alone	1	1.4	1	1.4	
Smoking					
No	68	98.6	70	98.6	0.984
Yes	1	1.4	1	1.4	

### Pain (VAS)

**Fig. 1:**
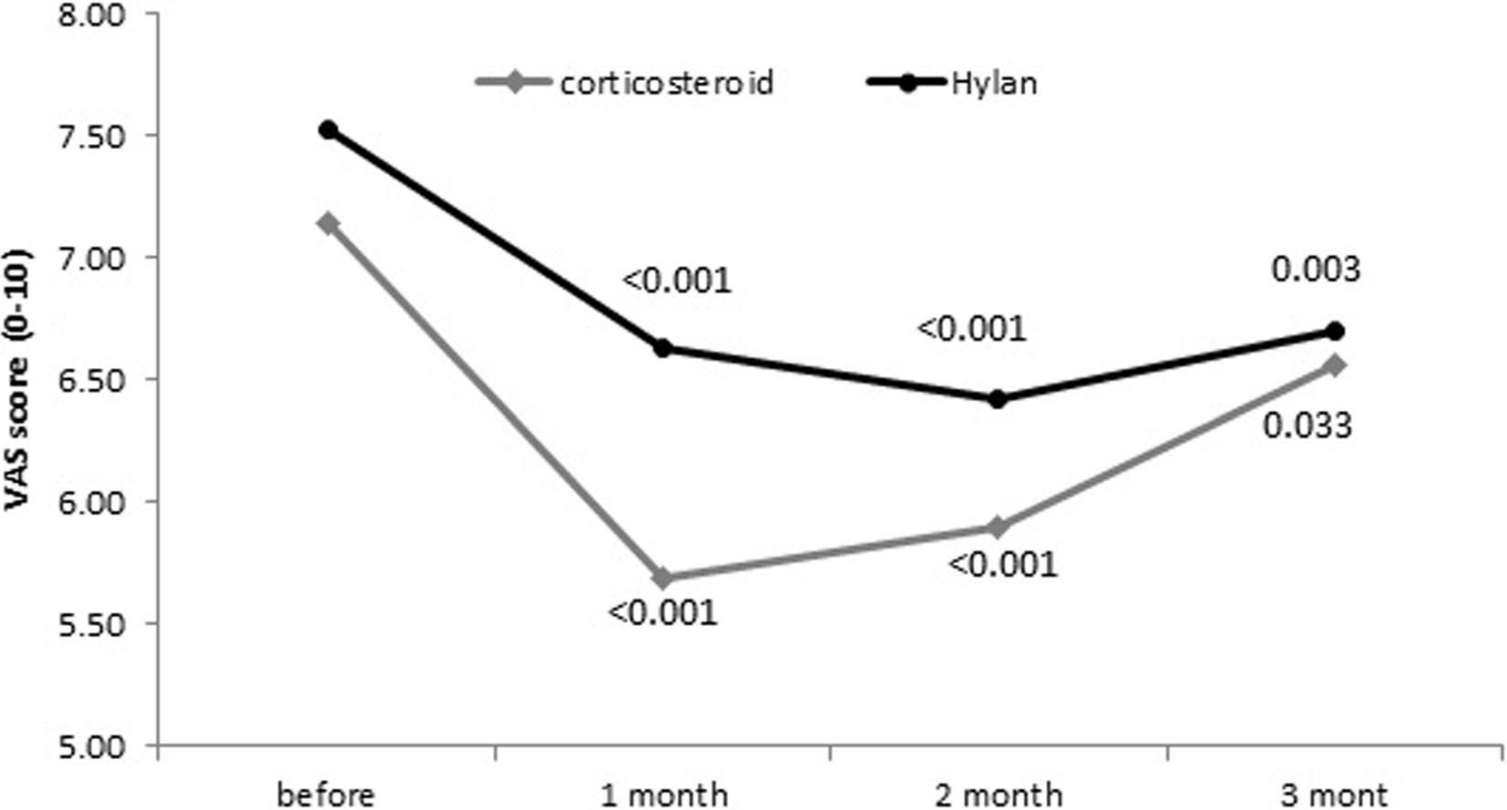
Comparison of pain trend in study groups

As shown in Fig. 1, before intervention pain score in corticosteroid group was 7.15 ± 2.01 same as Hylan group 7.52 ± 2.17 (P = 0.313). In corticosteroid group, pain at end of first month significantly decreased to 5.69 ± 2.33 (P < 0.001). At the end of second month, pain increased to 5.90 ± 2.33 but it was significantly lower than pain before intervention (P < 0.001). At end of third month, pain score increased to 6.56 ± 2.15 and it was not statistically different with primary pain (P = 0.200). In Hylan group at end of first month, pain significantly decreased to 6.63 ± 2.03 (P < 0.001). Unlike corticosteroid, at end of second month, pain continued its decreasing to 6.43 ± 2.01 (P < 0.001). At end of third month, pain score increased to 6.70 ± 2.01 but it was also significantly lower than primary pain (P = 0.020). The difference of pain between two groups was significant at end of first month (P = 0.018), but it was not significant at the end of second (P = 00.167) and third month (P = 0.720).

### WOMAC score

Pain (P = 0.093) and stiffness (P = 0.712) in corticosteroid group along with pain (P = 0.109) and stiffness (P = 0.112) in Hylan group were not statistically different before 3 months after intervention (Table 2). On the contrary, physical function problem significantly improved in both corticosteroid (P = 0.026) and Hylan (P = 0.043) groups.

**Table 2:** Comparison of WOMAC and KOOS Between Study Groups

	Corticosteroid (n = 69)		Hylan (n = 71)		P value ANOVA
	Mean	SD	Mean	SD	
WOMAC scores					
Pain before (0–20)	13.21	3.56	13.90	4.37	0.332
Pain after (0–20)	12.60	3.69	13.11	4.24	0.471
Stiffness before (0–8)	4.35	2.69	4.71	2.90	0.475
Stiffness after (0–8)	4.44	2.63	4.29	2.88	0.762
Physical function before (0–68)	35.98	11.36	35.90	12.38	0.970
Physical function after (0–68)	33.29	11.03	33.54	12.69	0.907
KOOS scores					
Pain before (0–100)	34.99	17.93	31.48	21.82	0.328
Pain after (0–100)	37.95	19.19	34.74	21.61	0.383
Symptoms before (0–100)	39.23	19.15	40.19	20.07	0.784
Symptoms after (0–100)	43.78	17.34	46.20	20.16	0.473
Daily activities before (0–100)	47.08	16.71	47.20	18.20	0.970
Daily activities after (0–100)	51.04	16.21	50.67	18.67	0.867

### KOOS score

Symptoms improved after 3 months in both corticosteroid (P = 0.010) and Hylan groups (P = 0.003). Besides, daily activity improved in both corticosteroid (P = 0.026) and Hylan groups (P = 0.046). On the contrary, pain did not decrease 3 months after intervention in both corticosteroid (P = 0.099) and Hylan groups (P = 0.170).

## Discussion

Osteoarthritis is a chronic disabling disease with morbidity and pain. Knee is a weight bearing joint frequently affected by degenerative processes which cause much disabilities. There are a few diseases modifying medical therapies for this disease condition. While primary treatment goals in knee OA include pain reduction improvement and improvement of joint mobility and function. Decreasing the progression of disease is an important secondary goal. Recent meta-analysis studies have argued that pharmacological interventions, to treat knee OA with oral NSAIDS, is inferior to intra-articular injections (Bannuru et al. 2015). Intra-articular injection of visco-supplementation with hyaluronic acid (HA) is a conservative intervention which is frequently administered with the hope of achievement of both primary and secondary therapeutic goals (Strand et al 2015).

Corticosteroids are other medications used as non-expensive pain modifying intra-articular injections (Bellamy et al. 2006), but there are no definite long-term benefits (Ray 2013). These two categories of intra-articular injections need to be clinically evaluated comparatively to assign their indications, contra-indications, cost-benefits and hence, to find their solid location in the algorithmic approach to the treatment of OA. Few studies have compared HA and CS (Bannuru et al. 2009). This can enrich the data regarding the use of non-surgical approaches to repair degenerated knee joints (Tiku and Sabaawy 2015). Thus, the aim of the present study was to determine which treatment method was more effective to pain alleviation and durability.

By using VAS, it was shown that both medications were equally effective in pain reduction in time points of first and second month after intervention. The effectiveness of pain reduction was more durable in HA group compared to CS. This was like what previous studies also provide (Ray 2013). At the end of the third month, pain score increased after decreasing at first and second month endpoints. At this point the score was not statistically different with the primary pain prior to intervention. It could be concluded that the duration of pain relief effectiveness is less than 3 months. On the contrary, the pain score remained significantly low after 3 months. In other words, the durability of efficacy of HA is more than 3 months and significantly longer compared to CS. Our result confirm the findings of Leighton et al., who reported the more durable effectiveness of HA compared to methylprednisolone (Leighton et al. 2014).

WOMAC score represented that pain and stiffness did not improve in neither groups at any time points after intervention (Table 2). However, physical function significantly improved in both groups. To our knowledge, this invaluable index has not been widely used in studies related to intra-articular injections (Vincent et al. 2013). However, this index is very appropriate in comparing the two injection methods. Besides, using KOOS score suggested that symptoms improved after 3 months in both corticosteroid and Hylan groups. Moreover, daily activity improved in both groups. This scoring system has provided a good clue to compare the clinical efficacy of each intervention (Peer and Lane 2013, van Meer et al. 2013).

Consequently, it is argued that the most important difference between the two interventions is the duration of effectiveness. Having compared to CS, HA is suggested to be superior in the duration of pain relief. We can propose that HA can be administered every 3 months intra-articular for knee joint OA. Therefore, it will be more convenient to use HA, when CS has to be injected every 2 months. It is probable that HA might be more cost-effective than CS although independent pharmacoeconomic studies are not sufficient. Our results present a more conservative treatment plan compared to the results of Abate et al. (2015), who have suggested a 4 months interval schedule for intra-articular injection of HA, while we have clearly shown that a 3 months schedule is superior. In other studies, gender-related difference has been reported for the effect of HA. We did not find this difference (Leopold et al. 2003).

The superiority of our study on others is its double blind method. Furthermore, we did not focus only on pain score. This study has used several scales to clarify any differences between the two intra-articular injections. Future studies with more extensive follow-ups are demanding and will be taken into account by our research group.
